# Can’t Stomach It: A Dilated Tale of Achalasia

**DOI:** 10.7759/cureus.113411

**Published:** 2026-07-26

**Authors:** Claire C Russell, Nicholas Diloreto, Caleb M Glover, Edward Cay, Dorian Jones

**Affiliations:** 1 Internal Medicine, McLaren Greater Lansing, Lansing, USA; 2 Gastroenterology, McLaren Greater Lansing, Lansing, USA

**Keywords:** aspiration hazard, esophageal achalasia, gastroesophageal surgery, gastrointestinal pathology, megaesophagus

## Abstract

Achalasia is a common esophageal motility disorder defined by impaired relaxation of the gastroesophageal junction (GEJ). Complications can occur secondary to achalasia, such as dysphagia, weight loss, chest pain, aspiration, and esophageal rupture. This report discusses a case of massive esophageal dilatation, also known as megaesophagus, in the setting of achalasia. This case highlights the complications secondary to achalasia, such as aspiration, pneumatic cysts, weight loss, dysphagia, and esophageal dilatation, and describes the multidisciplinary approach needed to manage patients with severe achalasia disease, with gastroenterology, pulmonology, and general surgery interventions required.

An 80-year-old male presented to the emergency department with a two-week history of progressively worsening dysphagia, accompanied by an inability to tolerate oral intake for several days. Initial computed tomography imaging revealed marked esophageal dilation with significant retained food debris, consistent with advanced achalasia. Additional imaging identified a right middle lobe pulmonary abscess, presumed secondary to aspiration. The patient had a known history of achalasia, previously managed with esophageal dilation, though they had not required intervention for several years prior to this hospitalization. The patient reported experiencing intermittent episodes of dysphagia since their last treatment; however, these episodes resolved spontaneously and did not prompt evaluation. In this instance, the prolonged duration and worsening of symptoms led to their presentation to the emergency department. The gastroenterology service was consulted for endoscopic evaluation and therapeutic dilation of the stricture. Due to the extent of the esophageal dilatation, measuring approximately 11 cm in diameter, fluoroscopic guidance was required to traverse the GEJ and enter the stomach. Endoscopy confirmed a significantly dilated esophagus with no food residue in the stomach with grossly normal-appearing gastric mucosa. A balloon dilation was successfully performed across the narrowed esophageal segment. Given the patient’s cachectic appearance and evidence of aspiration with pulmonary sequelae, general surgery was consulted for gastrostomy tube placement to minimize aspiration risk and provide nutritional support.

Achalasia is a primary esophageal motility disorder characterized by impaired lower esophageal sphincter (LES) relaxation and absent peristalsis of the esophageal body, leading to progressive obstruction and esophageal stasis. Over time, chronic retention of food and secretions can result in marked dilatation of the esophagus, sometimes referred to as megaesophagus when the diameter exceeds 6 cm. While esophageal dilation is a well-recognized complication, extreme dilation, exceeding 10 cm, is rare and may reflect long-standing disease. Prompt diagnosis and referral for cases of achalasia are critical; delays in treatment can lead to severe complications, including aspiration and malnutrition. Management may involve a multidisciplinary approach with gastroenterology and possible general surgery for pneumatic dilation, botulinum toxin injection, and myotomy evaluation, respectively. Timely recognition and coordination between inpatient and outpatient care teams is imperative for optimizing outcomes.

## Introduction

Achalasia is a rare primary esophageal motility disorder caused by degeneration of inhibitory neurons within the myenteric plexus, producing impaired relaxation of the lower esophageal sphincter (LES) and absent peristalsis of the esophageal body [1,2]. Its incidence is approximately one per 100,000 persons annually, and it typically presents with progressive dysphagia to solids and liquids, regurgitation, chest pain, or weight loss [3]. Radiologic severity is frequently graded by esophageal diameter, with dilation exceeding 6 cm commonly designated megaesophagus, although alternative thresholds, such as a diameter of 8 cm or more on barium study, have been proposed [4,5]. Most patients present earlier in the disease course, and only a minority progress to end-stage disease characterized by a markedly dilated, tortuous, aperistaltic esophagus [4,5]. Extreme dilation exceeding 10 cm is exceptional; as detailed in the Discussion, a focused review of the literature identified only isolated reports specifying such diameters. Long-standing achalasia carries risks of aspiration, recurrent respiratory infection, malnutrition, septic shock, airway compromise, and an increased incidence of esophageal carcinoma from chronic stasis and inflammation [2,4,6-8]. Definitive therapies include pneumatic dilation, laparoscopic Heller myotomy, and peroral endoscopic myotomy (POEM), with botulinum toxin injection reserved as a temporizing option; end-stage disease with massive dilation frequently requires multidisciplinary management and, in selected refractory cases, esophagectomy [6,9,10]. We present a case of extreme (11 cm) esophageal dilation complicated by an aspiration-related pulmonary abscess and malnutrition in an elderly man, and we describe the coordinated inpatient and outpatient management required. This report is prepared in adherence to the CARE (CAse REport) guidelines.

## Case presentation

Patient information

An 80-year-old man presented to the emergency department with a two-week history of progressively worsening dysphagia to solids and liquids and several days of near-complete inability to tolerate oral intake. He had a known history of achalasia previously treated with endoscopic dilation, with his most recent intervention several years earlier. Since that time, he had experienced intermittent, self-limited episodes of dysphagia that typically resolved within a week, and he had not maintained gastroenterology follow-up. The prolonged and progressive nature of his current symptoms prompted presentation.

Clinical findings

On arrival, the patient was afebrile, normotensive, and saturating normally on room air. He appeared cachectic; cardiopulmonary and abdominal examinations were otherwise unremarkable. His weight was 57.3 kg with a body mass index (BMI) of 19.8 kg/m². Admission vital signs and laboratory values are summarized in Table 1. Notable findings included leukocytosis, a low-normal albumin, mild hypophosphatemia, and a creatinine at the upper limit of the reference range that was elevated relative to the patient’s documented baseline (baseline creatinine: 0.6), consistent with prerenal acute kidney injury from poor oral intake. Hemoglobin was marginally below the reference range.

**Table 1:** Vital signs and laboratory results on admission

Test	Result	Units	Reference Range (Adult Male)
Heart Rate	94	beats per minute	60 – 100
Respiratory Rate	28	breaths per minute	12 – 20
White Blood Cell Count	16.79	×10⁹ per liter of blood	4.0 – 11.0
Hemoglobin	13.0	grams per deciliter of blood	13.5 – 17.5
Albumin	3.6	grams per deciliter of blood	3.5 – 5.0
Magnesium	1.9	milligrams per deciliter of blood	1.7 – 2.2
Phosphorus	2.4	milligrams per deciliter of blood	2.5 – 4.5
Creatinine	1.1	milligrams per deciliter of blood	0.7 – 1.2

Timeline

The chronology of the patient’s presentation, inpatient interventions, and outpatient follow-up is summarized in Table 2.

**Table 2:** Timeline of clinical events CT: computed tomography; LES: lower esophageal sphincter; PICC: peripherally inserted central catheter; POEM: peroral endoscopic myotomy.

Time Point	Event
Several years before admission	Established diagnosis of achalasia treated with multiple endoscopic dilations; no further intervention required for several years.
Interval (years)	Intermittent, self-limited episodes of dysphagia resolving within about a week; no gastroenterology follow-up maintained.
Two weeks before admission	Progressive dysphagia to solids and liquids.
Several days before admission	Near-complete inability to tolerate oral intake.
Day 0 (admission)	Presented to the emergency department. CT evidenced an 11 cm megaesophagus with retained food debris and right middle lobe fluid collections concerning for aspiration abscess. Admission labs (Table 1).
Hospital day 0–1	Fluoroscopy-assisted esophagogastroduodenoscopy with balloon dilation across the gastroesophageal junction; maintained nil per os, and general surgery was consulted for gastrostomy tube placement.
Hospital day 2-3	Infectious disease consultation for pulmonary abscesses; PICC line placement; intravenous antibiotics for polymicrobial pulmonary abscess. Gastrostomy was deferred to allow source control and stabilization.
Hospital day 5	General surgery placed a gastrostomy tube for enteral nutrition and aspiration-risk reduction.
Hospital day 7 (discharge)	Discharged to complete a 6-week intravenous antibiotic course; referred to a tertiary center for POEM evaluation.
Over >5 months of follow-up	Serial endoscopies; persistent retained esophageal debris repeatedly delaying POEM.
Most recent follow-up	POEM deferred after repeat endoscopy showed normal LES distensibility and diameter; gastroesophageal-junction ulcer and plaques biopsied (benign). Advanced to a soft diet with supplemental gastrostomy feeds; clinically stable.

Diagnostic assessment

**Figure 1:**
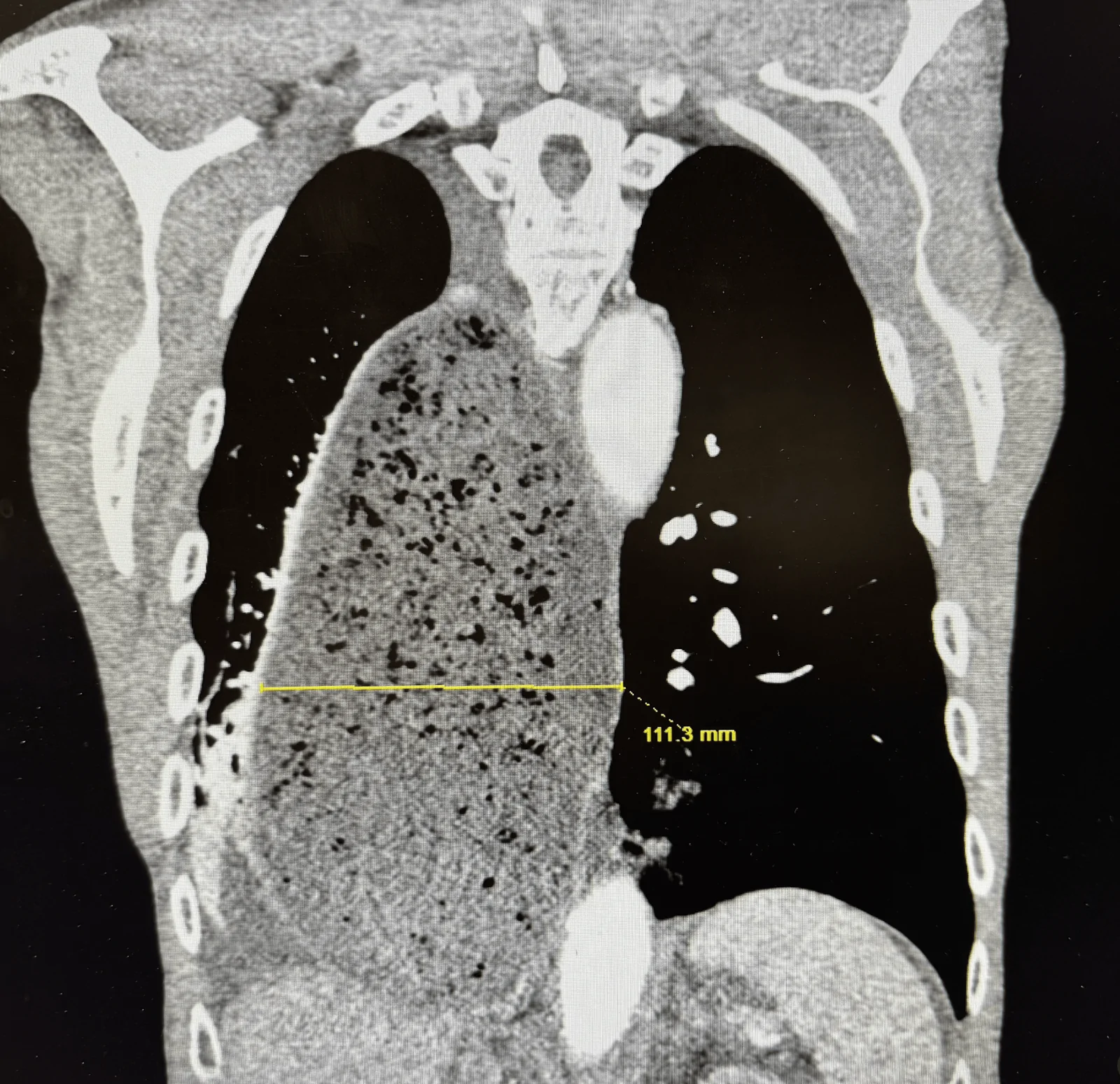
Coronal CT chest showing esophageal dilation (11 cm) with retained food debris

Contrast-enhanced computed tomography (CT) of the chest and abdomen demonstrated marked esophageal dilation measuring approximately 11 cm in maximal diameter with abundant retained food debris, consistent with advanced achalasia, together with multiple right middle lobe fluid collections concerning for aspiration-related pulmonary abscesses (Figure 1); subsequent cultures confirmed polymicrobial infection. Because of the volume of retained esophageal debris, the patient was intubated for airway protection prior to endoscopy. Esophagogastroduodenoscopy required fluoroscopic guidance to traverse the gastroesophageal junction (GEJ) and enter the stomach, and it confirmed a massively dilated esophagus containing food debris with grossly normal gastric mucosa and no gastric stasis. High-resolution manometry was not performed during this admission, given the acute presentation and retained debris; the diagnosis rested on the established clinical history together with characteristic imaging and endoscopic findings, with later assessment of LES distensibility.

Therapeutic interventions

Balloon dilation was performed across the GEJ with technical success. The patient was maintained nil per os. On the recommendation of the infectious disease service, a peripherally inserted central catheter was placed, and intravenous antibiotics were initiated for the pulmonary abscesses; gastrostomy placement was intentionally deferred several days to permit source control and clinical stabilization. Given his malnutrition and aspiration-related pulmonary complications, general surgery subsequently placed a gastrostomy tube to provide enteral nutrition and reduce aspiration risk. He completed a six-week course of intravenous antibiotics.

Nutritional assessment

The patient met the criteria for malnutrition. Applying the Global Leadership Initiative on Malnutrition (GLIM) framework, he fulfilled a phenotypic criterion (BMI 19.8 kg/m²), below the 22 kg/m² threshold applied to adults aged 70 years or older, in the context of reduced-intake-associated weight loss; together with etiologic criteria (markedly reduced oral intake over approximately two weeks and acute inflammation from the pulmonary abscess) [11]. Serum albumin (3.6 g/dL) was interpreted with caution, as it behaves as a negative acute-phase reactant and is an unreliable index of nutritional status during acute inflammation. Because prolonged near starvation combined with admission hypophosphatemia (2.4 mg/dL) conferred risk of refeeding syndrome, enteral nutrition was advanced cautiously with close biochemical monitoring, repletion of phosphorus, magnesium, and potassium, and thiamine supplementation. Formal quantification of muscle mass and additional visceral protein markers was not available [12].

Follow-up and outcomes

The patient was discharged to complete intravenous antibiotics and was referred to a tertiary care center for POEM evaluation. Over more than five months of follow-up with serial endoscopic evaluations, persistent retained esophageal food debris repeatedly delayed the planned procedure. POEM was ultimately deferred: repeat endoscopy demonstrated normal LES distensibility and diameter, indicating the absence of a residual outflow-obstructing target for myotomy. During evaluation, a deep ulcer and multiple white plaques proximal to the GEJ were biopsied; pathology was negative for malignancy and for fungal and viral infection. At the most recent follow-up, he had been advanced to a soft diet with supplemental gastrostomy feeds, was tolerating enteral nutrition, and remained clinically stable. No further POEM work-up or definitive surgical intervention was ongoing, as complete clearance of esophageal debris could not be achieved before evaluation.

## Discussion

Achalasia may progress to severe esophageal dilation, or megaesophagus, which predisposes to aspiration, malnutrition, and pulmonary complications [2,4]. The present case is distinguished by an exceptionally large dilation of 11 cm, complicated by a secondary lung abscess and malnutrition. Reports that specify an esophageal diameter at or above 10 cm are exceptional. In a focused (non-systematic) review of the English-language literature, the closest documented comparator identified was an 11.3 cm megaesophagus causing tracheal compression [13]; other "giant" cases are typically smaller, such as a 9.9 cm esophagus presenting with airway obstruction [14], and most reported megaesophagus cases fall within the 6-9 cm range. No registry or systematic tally quantifies cases exceeding 10 cm, partly because cross-sectional diameter is inconsistently reported and definitions of megaesophagus vary (a diameter greater than 6 cm radiologically versus 8 cm or more on barium study) [4,5]. This scarcity underscores the novelty of an 11 cm esophagus presenting with concurrent pulmonary abscess and nutritional compromise.

Management of advanced dilation is challenging. Balloon (pneumatic) dilation remains a standard intervention, although severe anatomic distortion may necessitate fluoroscopic guidance for safe passage across the GEJ, as in this patient [12]. POEM has emerged as an effective therapy even in markedly dilated esophagi, with single-operator and pooled data demonstrating high technical and clinical success [9,15]. Compared with pneumatic dilation, POEM offers greater durability, although postprocedural reflux and long-term outcomes remain considerations [10,16]. Esophagectomy is generally reserved for end-stage disease with a tortuous megaesophagus, persistent aspiration, or failure of less invasive therapy; it carries significant morbidity despite modern technique and remains a controversial last resort [9,17].

In this patient, POEM was deferred for two related reasons. First, myotomy, whether endoscopic or surgical, is directed at a non-relaxing, hypertensive, or spastic LES that generates outflow obstruction at the esophagogastric junction; candidacy therefore depends on demonstrating impaired junctional relaxation or reduced distensibility, typically by high-resolution manometry or endoluminal functional lumen imaging. On repeat evaluation, this patient’s LES demonstrated normal distensibility and diameter, indicating that no residual outflow-obstructing target remained to be divided, plausibly because prior dilation had already reduced junctional resistance. In this circumstance, the dominant problem was not junctional obstruction but the failure of an atonic, massively dilated, and tortuous conduit to empty by gravity, a mechanical limitation that myotomy does not correct, and after which iatrogenic reflux could be introduced without benefit. Second, persistent, retained food debris over more than five months prevented the mucosal clearance required for safe submucosal tunneling and increased procedural aspiration and infection risk, repeatedly precluding evaluation. Together, these factors excluded POEM, leaving esophagectomy, reserved as a last resort given its morbidity, or supportive enteral nutrition, the latter of which was pursued pragmatically through gastrostomy feeding.

Nutritional rehabilitation was central to this patient’s care. Beyond reducing aspiration risk, gastrostomy feeding provided a reliable route for caloric and protein delivery, enabling recovery from malnutrition and potentially improving candidacy for future intervention [9]. Because prolonged near-starvation with hypophosphatemia conferred refeeding-syndrome risk, feeds were advanced cautiously with electrolyte and thiamine repletion and close monitoring. Ongoing nutritional surveillance is essential, as long-standing disease frequently produces profound metabolic compromise. The combination of marked megaesophagus, an aspiration-related pulmonary abscess, and nutritional compromise in an elderly patient adds educational value to the existing literature on advanced achalasia [2].

This report has several limitations. As a single case, its findings may not be generalizable. High-resolution manometry was not obtained at this presentation, so the mechanistic characterization of the motility disorder is incomplete; the diagnosis relied on the established history, imaging, endoscopy, and subsequent assessment of LES distensibility. The nutritional assessment was constrained by the absence of a documented usual body weight, serial anthropometrics, muscle-mass quantification, and visceral protein markers, which limited formal grading of malnutrition severity. The literature comparison reflects a focused rather than a systematic review, and the true frequency of dilation exceeding 10 cm cannot be established, given the absence of a registry and inconsistent diameter reporting. Finally, follow-up was limited to several months, so long-term outcomes, including eventual esophagectomy candidacy and nutritional recovery, remain undetermined. 

## Conclusions

This case documents an exceptionally rare presentation of end-stage achalasia: an 11 cm megaesophagus complicated by an aspiration-related pulmonary abscess and malnutrition in an elderly patient. Two diagnostic lessons stand out. Severe anatomic distortion may require fluoroscopy-assisted endoscopy to traverse the GEJ safely, and assessment of LES distensibility by manometry or functional luminal imaging is decisive in determining myotomy candidacy; here, normal distensibility appropriately excluded POEM. Management reinforced the importance of coordinated, multidisciplinary care across gastroenterology, infectious disease, and surgery and of nutritional rehabilitation, with attention to refeeding risk, as a therapeutic priority rather than an afterthought. The principal clinical takeaway is preventive: because achalasia is progressive and can advance silently to extreme, poorly reversible dilation, patients warrant sustained longitudinal follow-up and timely referral for definitive therapy before end-stage megaesophagus and its aspiration- and nutrition-related complications develop.
